# Re-analysis of gene mutations found in pituitary stalk interruption syndrome and a new hypothesis on the etiology

**DOI:** 10.3389/fendo.2024.1338781

**Published:** 2024-02-23

**Authors:** Shengjie Wang, Qiaozhen Qin, Deyue Jiang, Yan Xiao, Lingtong Ye, Xiaoxia Jiang, Qinghua Guo

**Affiliations:** ^1^ Department of Endocrinology, The First Medical Center, Chinese PLA General Hospital, Beijing, China; ^2^ Beijing Institute of Basic Medical Sciences, Beijing, China

**Keywords:** pituitary stalk interruption syndrome, gene sequencing, enrichment analysis, traction during delivery, etiological hypothesis

## Abstract

**Background:**

Pituitary stalk interruption syndrome (PSIS) is a complex clinical syndrome characterized by varied pituitary hormone deficiencies, leading to severe manifestations across multiple systems. These include lifelong infertility, short stature, mental retardation, and potentially life-threatening pituitary crises if not promptly diagnosed and treated. Despite extensive research, the precise pathogenesis of PSIS remains unclear. Currently, there are two proposed theories regarding the pathogenic mechanisms: the genetic defect theory and the perinatal injury theory.

**Methods:**

We systematically searched English databases (PubMed, Web of Science, Embase) and Chinese databases (CNKI, WanFang Med Online, Sinomed) up to February 24, 2023, to summarize studies on gene sequencing in PSIS patients. Enrichment analyses of reported mutated genes were subsequently performed using the Metascape platform.

**Results:**

Our study included 37 articles. KEGG enrichment analysis revealed mutated genes were enriched in the Notch signaling pathway, Wnt signaling pathway, and Hedgehog signaling pathway. GO enrichment analysis demonstrated mutated genes were enriched in biological processes such as embryonic development, brain development, axon development and guidance, and development of other organs.

**Conclusion:**

Based on our summary and analyses, we propose a new hypothesis: disruptions in normal embryonic development, partially stemming from the genetic background and/or specific gene mutations in individuals, may increase the likelihood of abnormal fetal deliveries, where different degrees of traction during delivery may lead to different levels of pituitary stalk interruption and posterior lobe ectopia. The clinical diversity observed in PSIS patients may result from a combination of genetic background, specific mutations, and variable degrees of traction during delivery.

## 1 Introduction

Pituitary stalk interruption syndrome (PSIS) encompasses a constellation of clinical manifestations arising from the absence or interruption of the pituitary stalk, leading to impaired transport of hormone-releasing hormones secreted by the hypothalamus to the pituitary (1, 2). Diagnosis of PSIS primarily relies on pituitary magnetic resonance imaging (MRI), revealing characteristic features such as hypoplastic anterior pituitary, interrupted or absent pituitary stalk, and ectopic posterior pituitary (3–6). PSIS patients exhibit varying degrees and types of anterior pituitary hormone deficiencies, often accompanied by midline structural abnormalities. PSIS can lead to severe and unfavorable clinical outcomes, including lifelong infertility, stunted growth, cognitive impairment, and even life-threatening pituitary crises (7, 8).

The pathogenesis of PSIS remains poorly understood, and current understanding is based on two prevailing theories. The first theory posits that perinatal injury during delivery is a contributing factor to PSIS. This hypothesis stems from retrospective analyses demonstrating a notably high incidence of breech delivery and perinatal events among PSIS patients. Literature reported a rate of 18% breech delivery among PSIS patients (9), whereas our study showed a substantially higher rate of 45.8% in PSIS patients (10). Nevertheless, a subset of PSIS patients presents neither perinatal events nor breech presentation. Furthermore, some PSIS patients have a family history, and some patients exhibit structural dysplasia in the central nervous system midline. As a result, a growing number of researchers speculate an association between PSIS pathogenesis and congenital genetic defects. However, genetic investigations pertaining to the pathogenesis of PSIS only yielded limited progress, with causative genes identified in less than 5% of PSIS patients (9). Furthermore, the relationship between gene mutations and PSIS remains unclear in the majority of reports. As such, comprehensive research efforts are warranted to decipher the pathogenesis of PSIS.

This article presents a comprehensive compilation, consolidation, and synthesis of gene sequencing data obtained from PSIS patients across multiple research teams. Additionally, we performed gene ontology (GO) and Kyoto Encyclopedia of Genes and Genomes (KEGG) enrichment analyses on the identified genes to identify potential pathogenic genes associated with PSIS and to propose novel research directions. Additionally, based on our summary and analyses, we propose a novel etiological hypothesis through logical arguments.

## 2 Methods

### 2.1 Literature search strategy

A systematic literature search was conducted in English databases (PubMed, Web of Science, Embase) and Chinese databases (CNKI, WanFang Med Online, Sinomed), spanning from the inception of the databases up to February 24, 2023. The search strategy employed the following query: “[(pituitary stalk interruption syndrome) OR (pituitary stalk transection syndrome)] OR (pituitary stalk truncation syndrome)”. Fine adjustments were made to the search strategy to align with the specific requirements of each database. Furthermore, the reference lists of included articles were manually screened to ensure the inclusion of all relevant studies.

### 2.2 Literature screening and data extraction

Two independent reviewers, the first and second authors, conducted the literature screening, data extraction, and cross-verification. After removing duplicates using NoteExpress software, articles pertaining to gene sequencing in PSIS patients were identified by carefully reviewing the titles and abstracts of the remaining articles. Subsequently, relevant data were extracted from the full texts, including the year of publication, first author’s name, the number of PSIS patients who underwent gene sequencing, the number of PSIS patients with gene mutations, gene sequencing methods employed, mutated genes reported in PSIS patients, functional verification procedures, breech delivery rate, family history, extra-pituitary malformation rate, sex ratio and hormone deficiency rate. Considering the retrospective nature inherent to our study design, we employed the abbreviation ND to indicate “Not Documented” for information lacking documentation in the existing literature. Significantly, in the calculation of rates or ratios, a principled exclusion of such undocumented information was implemented. This decision was guided by the fundamental principle of balancing the completeness and reliability of the analysis results within the confine of available recorded data.

### 2.3 Enrichment analyses of mutated genes reported in PSIS patients

For the mutated genes reported in PSIS patients, enrichment analyses were performed using the Metascape platform (11, 12), which encompassed pathway and process enrichment analyses, specifically focusing on KEGG Pathway, GO Biological Processes, GO Cellular Components, and GO Molecular Functions. The protein-protein interactions (PPI) among the input genes were extracted from a reliable PPI data source, thereby forming a PPI network (13). Subsequently, the Molecular Complex Detection (MCODE) algorithm was applied to identify densely connected protein neighborhoods within this network (14). Additionally, heatmaps were generated using an online platform (https://www.bioinformatics.com.cn) for data analysis and visualization. The resulting bubble charts displayed specific biological processes, cellular components, molecular functions, or pathways on the vertical axis. The horizontal axis denoted the enrichment ratio, while the color and size of the bubbles reflected the associated *P* value and the number of enriched genes, respectively.

## 3 Results

### 3.1 Overview of the literature search results

**Figure 1:**
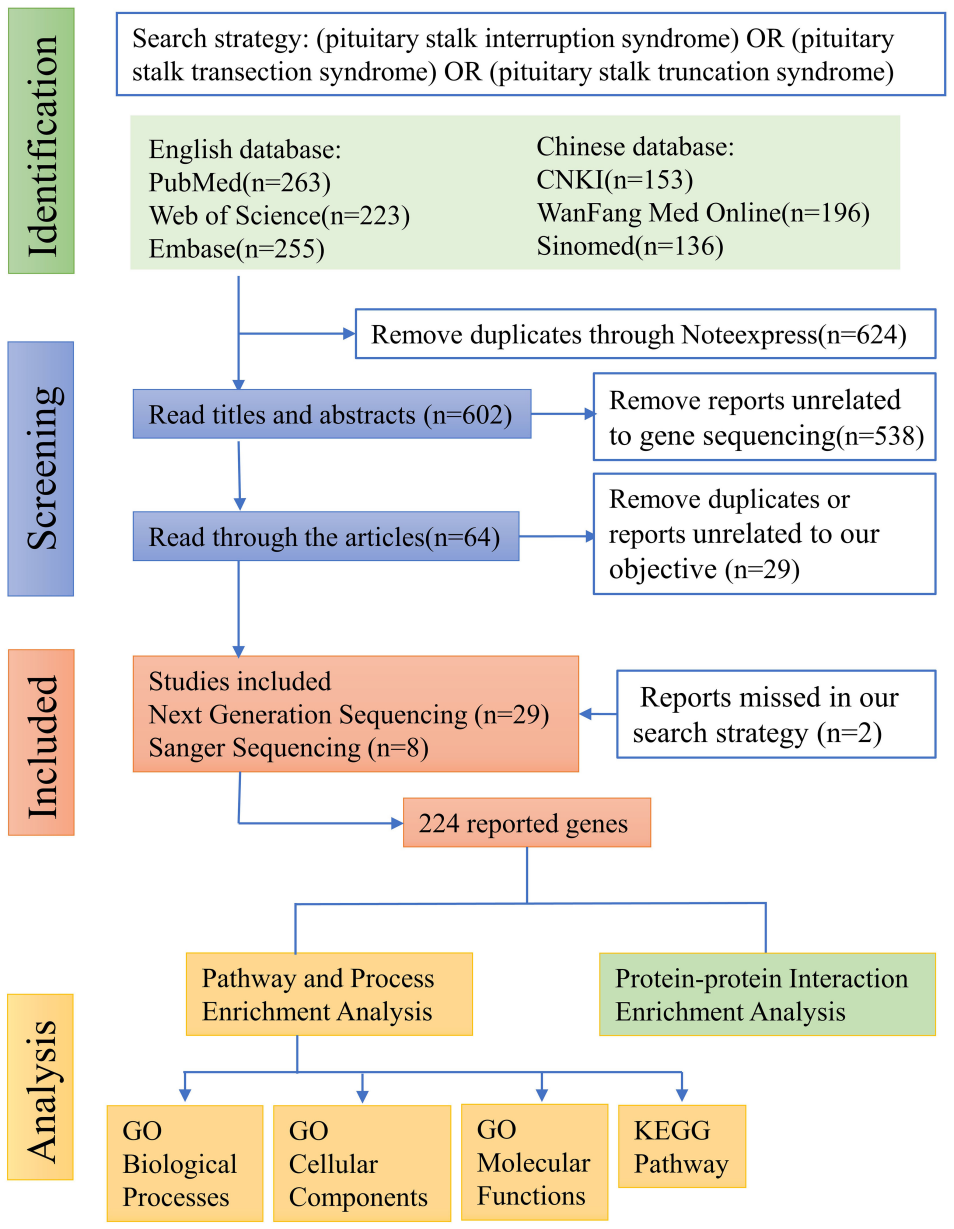
Flow diagram of literature screening. For details, please refer to “2. Methods”.

The literature search, following the aforementioned search strategy, yielded a total of 263 records from PubMed, 223 from Web of Science, 255 from Embase, 153 from CNKI, 196 from WanFang Med Online, and 136 from Sinomed, respectively. Subsequently, a total of 624 duplicate records were identified and removed through NoteExpress literature management software. After carefully reviewing the titles and abstracts of the remaining 602 records, we identified 64 articles that reported mutated genes in PSIS patients. Further scrutiny of the full texts of these 64 articles resulted in the exclusion of 29 articles that were either duplicate or not meeting with our research objective. Additionally, through rigorous reference tracking, we identified and included 2 articles that had initially been overlooked. Consequently, 37 articles were included in our analysis, among which 29 articles employed next-generation sequencing techniques in PSIS patients, while 8 articles only reported the application of Sanger sequencing (**Figure 1**). However, subsequent functional validation was conducted in only 10 articles, suggesting the existence of numerous candidate pathogenic mutations that require further functional verification (**Table 1**). By summarizing the mutated genes reported in these studies and removing duplicates, we identified a total of 224 reported genes (**Supplementary Table 2**), based on which we conducted subsequent enrichment analysis.

**Table 1:** The detailed information of 37 included articles.

Year	FA	N1	N2	SM	Mutated Genes	FV	BP	FH	EM	Gender	GHD	ACTHD	TSHD	FSH/LHD	DI
2023	Bando, H. (15)	1	1	Exome sequencing	SIX3, POU1F1	Y	0	N	0/1	F1	1/1	1/1	1/1	0/1	0/1
2022	Silva, T.S. (16)	78	15	WES	TOMM70, RBBP9, CDC16, RIPPLY2, CDK5, PINLYP, COX11, HESX1, HSD11B2, KCNMB2, PPP2R5D, ROBO1	N	6/78	0/78	17/78	58M:20F	78/78	34/78	37/78	30/78	0/78
2021	Ji, W.^	10	10	WES	AR, MAGEL2, MTHFR, PKD1, EOMES, GPR101, BMP2, CFTR, BRIP1, ZSWIM6, RELN, KAT6A, BMP4, WASHC5, BRCA2, CDH23, KLHL10, NCOR2, KAT6B, MLH1, NSD1, PDGFRB, AVP, DCHS1, GANAB, HHAT, TBX2, TUBB3, CHD7, KMT2A, IGSF1, PKD2, SIX5	N	0/10	0/10	1/10	8M:2F	10/10	8/10	9/10	10/10	2/10
2021	Gregory, L. C. (17)	2	2	PCR-sanger, gene panel, exome sequencing	OTX2	Y	0/2	N	1/2	M1:F1	2/2	1/2	1/2	1/2	0/2
2021	Obara-Moszynska, M. (18)	1	1	WES	CDON	N	0/1	N	1/1	F1	1/1	1/1	1/1	1/1	0/1
2021	Kaygusuz, S. B. (19)	2	2	NGS	FOXA2	Y	0/2	N	2/2	M2	2/2	2/2	2/2	0/2	1/2
2020	Brauner, R. (20)	52	39	WES	PROP1, IL17RD, SMARCA2, GLI3, SHH, CHD7, NBAS, KIAA0556, ROBO1, FANCA, SEMA3E, SLX4, CFTR, WDR11, PMM2, DNMT1, NSMF, ARID1B, VPS13B, LHX9, INPP5E, BMP4, CDON, GLI2, PTCH1, FANCG, FANCD2, RAF1, CCDC141, TBX19, PRMT7, NKX2-1, SOX11, TGIF1, FANCC, FGFR3, CSPP1, WT1, FANCE, DHCR7, ZNF423, FSHR, CEP120, SLIT2, CC2D2A, KISS1R, GATA5, HESX1	N	ND	2families/52individuals*	17/37	33M:19F	ND	ND	ND	ND	1/52
2020	Fang, X. (21)	59	50	WES	PTCH1, PTCH2, AHI1, ATR, CHD7, GLI2, PRKAR2A, TCTN1, CAD, CDON, CEP152, CEP290, DHCR24, DMXL2, FREM1, GLI1, GPSM2, ISPD, NIN, ROBO2, SIX4, SLIT2, SPG11, STK36, WDR11, ASPM, CENPJ, CEP41, CREBBP, DIS3L2, DISC1, DSC2, EGR4, GH1, GNAS, HHAT, KIF7, LHX4, LRP2, MAPK3, MARCKS, MYH10, NPHP1, NSD1, OTUD4, PCSK1, POMGNT1, PRKAR2B, PSEN1, RNF111, SMO, STIL, TACR3, TBC1D32, VIPR2, WNT5A, ZEB2, ZNF423	N	40/59	ND	ND	51M:8F	59/59	54/59	56/59	59/59	1/59
2020	Lodge, E. J. (22)	28	6	WES	DCHS2, FAT2	Y	ND	ND	0/6	5M:1F	6/6	2/6	3/6	ND	ND
2020	David, O. (23)	2	2	WES	TTC26	N	0/2	N	2/2	M1:F1	2/2	2/2	2/2	0/2	0/2
2020	Liu, Z. (24)	1	1	WES	ROBO1	N	0/1	Y	1/1	M1	1/1	1/1	1/1	0/1	0/1
2020	Wang, C. Z. (25)	1	1	WGS	MUC12/NBPF9/TYW1B	Y	1/1	N	1/1	M1	1/1	1/1	1/1	0/1	0/1
2020	Castets, S. (26)	3	1	NGS	FOXL2	N	0/3	N	3/3	F1:ND2	3/3	2/3	2/3	2/3	0/3
2020	Demiral, M. (27)	1	1	NGS	GLI2	N	0/1	N	1/1	M1	1/1	1/1	1/1	1/1	0/1
2019	Correa, F. A. (28)	11	11	WES	LCMT1, PCDHB14, ATXN1, ATXN7, KRT18, SYNE1, WDR27, CC2D2A, HAUS5, IFT140 and ELF4	N	ND	ND	ND	ND	ND	ND	ND	ND	ND
2019	Wang, D. (29)	1	1	WES	COL1A1, COL1A2	N	1/1	N	1/1	M1	1/1	1/1	1/1	1/1	0/1
2019	Wang, C. Z. (30)	2	2	WES	MUC4, NBPF10	N	1/2	N	0/2	M2	2/2	1/2	1/2	2/2	0/2
2019	Dateki, S. (31)	1	1	WES	ROBO1	N	0/1	N	1/1	M1	1/1	1/1	1/1	1/1	0/1
2019	Berkun, L. (32)	1	1	WES	MAPRE2, CDON	N	0/1	N	1/1	F1	ND	ND	ND	ND	ND
2018	Zwaveling-Soonawala, N. (33)	20	18	WES	ARNT2, BMP4, CHD4, GLI3, SIX6, IGSF1, GLI2, B9D1, CHD7, FGF8, NR0B1, OTUD4, PROK2, TACR3, ASPM, CC2D2A, DHCR7, INPP5E, NDE, RELN, SLC12A6, CCDC88C, DCHS1, KAT6A, KIF14, ROBO2	N	ND	0/20	ND	14M:6F	20/20	19/20	20/20	17/20	0/20
2018	Su, X.^	1	1	WES	CDC27, NF1, USP9X	Y	0/1	N	0/1	F1	1/1	0/1	0/1	0/1	0/1
2017	Bashamboo, A. (34)	25	5	WES	ROBO1	N	ND	2families/25individuals*	4/5	3M:2F	5/5	1/5	2/5	1/5	ND
2017	Guo, Q.H. (10)	24	22	WES	DAAM1, DVL1, GLI3, LRP2, ZIC2, AXIN1, PRICKLE2, NCOR2, NOTCH3, DLL1, JAG1, NCOR2, NKD2, JAG1, LRP6, BMP6, CTBP2, GLI3, RBPJ, MYC, NKD2, MMP7, NCOR2, BMP8B, FRAT1, ZIC2, FZD1, LRP2, BMP6, NCOR2, ZIC2, DVL1, PSEN1, MAML3, ADAM17, ROCK2, HHIP, DLL4, CREBBP, FZD8, PLCB2, PSEN2, BMP6, MAML3, GLI1, NKD2, NCOR2, LFNG, NFATC2, PPP2R5B, NCOR2, CREBBP, STK36, NKD2, CHP2, CER1, NKD2, NUMBL, DLL4, ZIC2, PSEN2, NFATC1, NCOR2, NKD2, MAML3	N	11/24	0/24	ND	22M:2F	24/24	17/24	13/24	17/24	ND
2017	Zheng, J. J.^	85	36	WES	FGFR2, GLI3, NFKB2, WDR11, GLI2, NOTCH2, LHX4, NOTCH3, CDON, SEMA3A, CHD7, POU1F1, PTCH1, GLI1, GATA2, ARNT2, FGFR1, WNT4, TBX19, SIX4, BMP4, POMC, OTX1, LHX1, PROKR2, MSX1	N	73/86	1family/85individuals	4/86	75M:11F	86/86	42/86	69/86	84/86	5/86
2017	McCormack, S. E. (35)	1	1	WES	PROKR2, WDR11	Y	0/1	N	0/1	M1	1/1	1/1	1/1	1/1	0/1
2017	Zwaveling-Soonawala, N. (36)	1	1	WES	KAT6A, BMP4, GLI3	N	0/1	N	1/1	F1	1/1	1/1	1/1	0/1	0/1
2016	Bashamboo, A. (37)	1	1	WES	CDON	N	1/1	N	0/1	F1	1/1	1/1	1/1	0/1	0/1
2015	Karaca, E. (38)	2	2	WES	GPR161	N	0/2	1family	2/2	F2	2/2	0/2	1/2	0/2	1/2
2014	Izumi, Y. (39)	1	1	NGS	WDR11	N	0/1	N	0/1	M1	1/1	0/1	0/1	1/1	0/1
2013	Yang, Y. (40)	33	1	PCR-sanger	HESX1	N	50/58	0/58	2/58	51M:7F	58/58	45/58	25/58	49/58	1/58
2013	Tatsi, C. (41)	30	2	PCR-sanger	TGIF, SHH	N	ND	ND	3/30	ND	ND	ND	ND	ND	ND
2012	Reynaud, R. (42)	72	4	PCR-sanger	PROKR2, HESX1	Y	ND	1family/72individuals	ND	39M:33F	ND	ND	ND	ND	ND
2011	Reynaud, R. (9)	69	3	PCR-sanger	HESX1, LHX4	N	11/61	1family/69individuals	24/83	58M:25F	83/83	56/83	66/83	54/83	ND
2010	Tatsi, C. (43)	25	1	PCR-sanger	SHH	N	ND	ND	ND	ND	ND	ND	ND	ND	ND
2008	Castinetti, F. (44)	136	2	PCR-sanger	LHX4	Y	ND	ND	ND	ND	135/136	65/136	88/136	60/136	ND
2006	Reynaud, R. (45)	39	1	PCR-sanger	LHX4	N	ND	1family/39individuals*#*	ND	ND	ND	ND	ND	ND	ND
2001	Machinis, K. (46)	2	2	sanger	LHX4	Y	ND	1family*#*	2/2	M1:F1	2/2	2/2	2/2	1/2	0/2
Total	37 reports	824	252	29NGS:8Sanger	224 genes	10/37	195/402	6families/560individuals	92/421	431M:148F	591/592	362/592	409/592	391/592	12/390

FA, First Author; N1, Number of patients for sequencing; N2, Number of patients with gene mutation; SM, Sequencing Method; FV, Functional Verification; BP, Breech Presentation; FH, Family History; EM, Extra-pituitary Malformation; NGS, Next Generation Sequencing; WES, Whole Exome Sequencing; WGS, Whole Genome Sequencing; PCR, Polymerase Chain Reaction; GHD, Growth Hormone deficiency; ACTHD, Adrenocorticotropin deficiency; TSHD, Thyroid-stimulating hormone deficiency; FSH/LHD, Follicle stimulating hormone/Luteinizing hormone deficiency; DI, Diabetes Insipidus; N, Not; Y, Yes; M, Male; F, Female; ND, Not documented; *means the same two families; # means the same family; ^ means reports from Chinese thesis.

### 3.2 Clinical characteristics of PSIS patients in the included articles

By summarizing these 37 articles, a total of 824 PSIS patients were included, with 252 of them found to carry gene mutations. By excluding the undocumented information, the male-to-female ratio was determined to be 3.12:1(431males/148 females). Breech presentation was observed in 48.5% of cases (195/402). The proportion of PSIS patients with a family history was 1.1% (6 families/560 individuals), and extra-pituitary abnormalities were present in 21.9% of cases (92/421). The rate of growth hormone deficiency (GHD), adrenocorticotropic hormone deficiency (ACHD), thyroid-stimulating hormone deficiency (TSHD), follicle-stimulating hormone/luteinizing hormone deficiency (FSH/LHD), and diabetes insipidus was 99.8% (591/592), 61.1% (362/592), 69.1% (409/592), 66.0% (391/592), and 3.1% (12/390), respectively (**Table 1**).

In the studies only using Sanger sequencing, mutations in 5 genes (HESX1, TGIF, SHH, PROKR2, LHX4) were identified in PSIS patients. While the remaining 219 genes were identified by next generation sequencing, including whole-exome sequencing and whole-genome sequencing (**Table 1**).

During the literature screening process, 6 articles describing chromosomal variations in PSIS patients were identified (**Table 2**). However, due to the inability to confirm the exact gene changes caused by chromosomal deletions or duplications, these 6 articles were not included in our analysis.

**Table 2:** The detailed information of 6 articles describing chromosomal variations in PSIS patients.

Year	FA	N3	Methods	Type	Size (Mb)	classification	region	FH	Perinatal events	Title
2023	Correa-Silva, S. R. (47)	2	array-CGH	Del	1.8	Pathogenic	17q12	*N*	*N*	Copy number variation in pituitary stalk interruption syndrome, A large case series of sporadic non-syndromic patients and literature review
				Del	15	Pathogenic	18p11.32 p11.21	N	N	
2021	Mnif-Feki, M. (48)	1	FISH	ND	ND	ND	ND	N	N	Occurrence of Hypopituitarism in Tunisian Turner Syndrome patients, familial versus sporadic cases
2016	Bartkevica, L. (49)	1	FISH	Del	ND	ND	13q12.3-q14.3	ND	N	13q deletion and pituitary stalk interruption in bilateral retinoblastoma
2014	Vetro, A. (50)	1	array-CGH; FISH	Gain	9.6	ND	2p25	N	N	Severe growth hormone deficiency and pituitary malformation in a patient with chromosome 2p25 duplication and 2q37 deletion
				Del	7.3	ND	2q37	N	N	
2013	Tatsi, C. (41)	1	Conventional chromosome analysis	Del	ND	ND	18p	ND	ND	Pituitary stalk interruption syndrome and isolated pituitary hypoplasia may be caused by mutations in holoprosencephaly-related genes
2011	El, C. S. (51)	1	array-CGH; FISH	Del	493kb	ND	17q21.31	ND	ND	17q21.31 microdeletion in a patient with pituitary stalk interruption syndrome

N3, Number of patients with chromosome variation; FH, Family History; N, Not; ND, Not documented; FISH, Fluorescence *in situ* hybridization; array-CGH, array comparative genomic hybridization.

When summarizing the reported genes, we observed that GLI2, PTCH1, ROBO1, CHD7, and CDON genes were mutated in 15, 13, 12, 12, and 10 patients, respectively. Among the 224 mutated genes reported in 252 PSIS patients, mutations in 141 genes were reported only once, mutations in 42 genes were reported twice, and mutations in 22 genes were reported three times (**Supplementary Table 2**). This pattern underscores the sporadic nature of these gene mutations in PSIS patients, highlighting the necessity for further in-depth research.

### 3.3 Pathway and process enrichment analysis results

**Figure 2:**
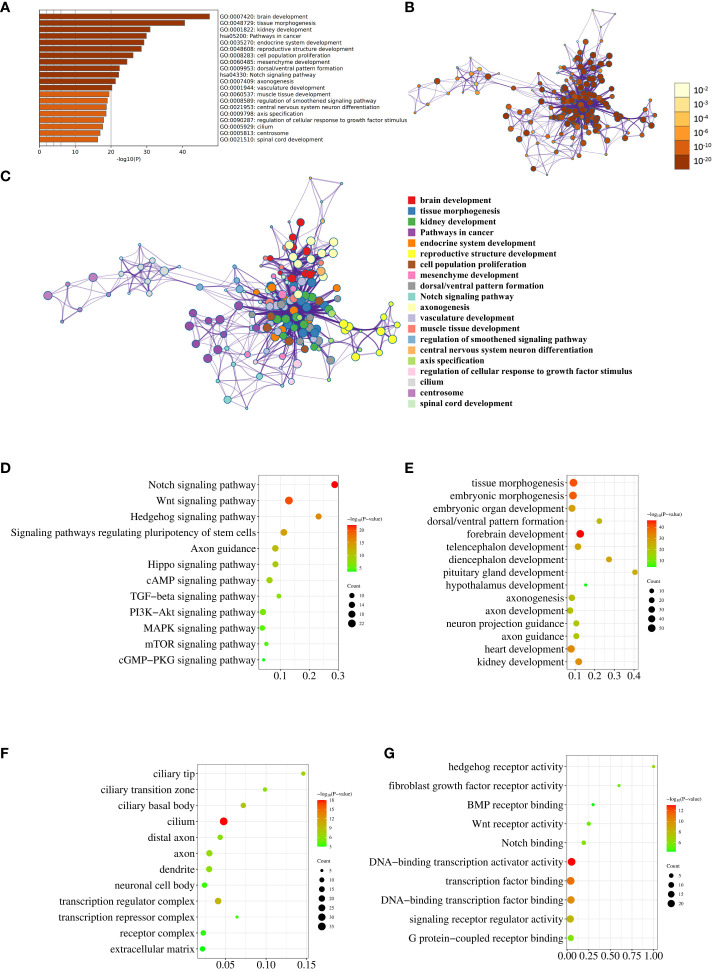
Pathway and process enrichment analysis. **(A)** The bar graph displays enriched terms across input gene lists, colored by *P* value. **(B)** The network illustrates enriched terms colored by *P* value, with terms containing more genes typically having a more significant *P* value. **(C)** The network represents enriched terms colored by cluster ID, where nodes sharing the same cluster ID are generally close to each other. **(D–G)**. The enrichment analysis results of the input genes in KEGG Pathways **(D)**, GO Biological Processes **(E)**, GO Cellular Components **(F)**, and GO Molecular Functions **(G)** are visually depicted through the utilization of bubble charts.

Top 20 enriched terms across the input gene list were colored by *P* value (**Figure 2A**). Network of enriched terms was colored by *P* value, where terms containing more genes tend to have a more significant *P* value (**Figure 2B**). Network of enriched terms was colored by cluster ID, where nodes that share the same cluster ID were typically close to each other (**Figure 2C**). This set of three graphs provides a general overview of pathway and process enrichment analysis.

Subsequently, we conducted individual analyses to evaluate the enrichment of reported genes in KEGG pathways, GO biological processes, GO cellular components, and GO molecular functions. The KEGG enrichment analysis revealed that 17, 22, 13, 16, and 15 genes were enriched in the Notch signaling pathway, Wnt signaling pathway, Hedgehog signaling pathway, Signaling pathways regulating pluripotency of stem cells, and Axon guidance, respectively (**Figure 2D**; **Supplementary Table 1**). Notably, these results are in agreement with the conclusion of our previous sequencing study (10). The enrichment of 224 genes in biological processes related to embryonic development, brain development, axon development and guidance, and development of other organs was illustrated in **Figure 2E** using a bubble chart. The GO Cellular Components enrichment analysis revealed that the genes were mainly enriched in cell components such as cilium, axons, dendrites, and cytoplasm, as shown in **Figure 2F**. The GO Molecular Functions enrichment analysis indicated that the genes were mainly enriched in molecular functions related to hedgehog receptor activity, fibroblast growth factor receptor activity, BMP receptor binding, Wnt receptor activity, Notch binding, and transcriptional regulation, as depicted in **Figure 2G**. The detailed genes enriched in various pathways and processes that match **Figures 2D–G** were listed in **Supplementary Table 1**.

### 3.4 Protein-protein interaction enrichment analysis results

**Figure 3:**
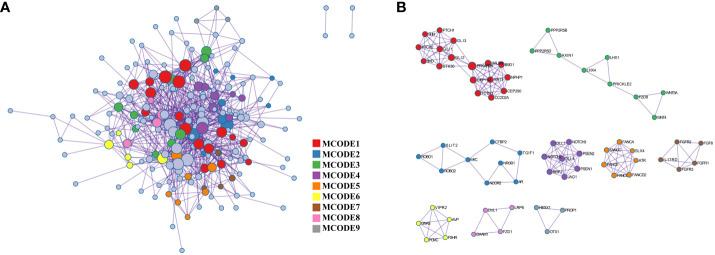
Protein-protein interaction enrichment analysis. **(A)** The protein-protein interaction network illustrates the intricate patterns of interactions between proteins, aiding in our understanding of how proteins are interconnected and regulated within cellular biology processes. **(B)** The MCODE components are modular protein interaction clusters that have been pinpointed through the analysis of gene lists, facilitating the recognition of key gene sets that play crucial roles in biological processes.

The PPI network, as shown in **Figure 3**, was visualized using Metascape, which applies the MCODE algorithm to detect densely connected regions. MCODE1-9 were displayed in different colors (**Figure 3A**), and within the same MCODE, connecting lines signified physical interactions between the proteins at both ends (**Figure 3B**). In the largest cluster identified as MCODE1, the proteins were predominantly enriched in the Hedgehog signaling pathway and in cellular ciliary components (**Figure 3**; **Supplementary Table 1**). MCODE3 and MCODE4 depicted protein-protein interactions in the Wnt and Notch signaling pathway, respectively. By visualizing the network, one might gain a more comprehensive understanding of the intricate molecular interactions underlying the pathogenesis of PSIS. These MCODE components provided potential targets for further investigation and might uncover key molecular mechanisms underlying the development and progression of PSIS.

## 4 Discussion

### 4.1 Two primary etiological hypotheses of PSIS currently

The etiology of PSIS remains enigmatic, and two primary etiological hypotheses have emerged: the perinatal injury theory and the congenital genetic defect theory.

Evidence supporting the perinatal injury theory includes: 1) There is a significantly higher prevalence of perinatal adverse events, such as breech delivery, cesarean section, and neonatal distress, in PSIS patients compared to the general population (40%-90% vs 4%) (52–54). 2) The prevalence rate of breech delivery was significantly higher in the PSIS-CPHD subgroup [combined pituitary hormone deficiency (CPHD) patients diagnosed with PSIS] than in the POU1F1/PROP1-CPHD subgroup (CPHD patients carrying POU1F1/PROP1 pathogenic mutations) (44.4% vs 5.5%, P=0.004) (55). Yang et al. also noted that PSIS patients had a higher rate of breech delivery than patients with normal pituitary stalk (40). 3) A patient diagnosed with PSIS was born in the breech position, while his monozygotic twin brother was born in the normal position with a normal pituitary stalk (29). However, evidence against the perinatal injury theory includes: 1) Breech presentation is the most common abnormal fetal presentation, accounting for 3% to 4% of full-term deliveries. However, not all breech deliveries lead to PSIS, and the incidence of PSIS is much lower than the rate of breech deliveries; 2) Some PSIS patients were born with a normal fetal presentation or without adverse perinatal events; 3) Some PSIS patients had a family history of the condition; 4) A certain proportion of patients had structural dysplasia of central nervous system midline.

Currently, the congenital genetic defect hypothesis is also gaining support among scholars as a plausible explanation for the pathogenesis of PSIS. Several lines of evidence are supportive of this hypothesis. Firstly, the occurrence of PSIS in patients born in cephalic presentation, without any apparent perinatal injuries, suggests that the etiology of PSIS cannot be solely attributed to perinatal injury. This observation argues against perinatal factors in the development of PSIS. Secondly, the presence of family history in some PSIS patients provides further support for the involvement of genetic factors. The familial clustering of the condition implies the existence of inherited genetic variations that contribute to the development of PSIS. Thirdly, gene sequencing techniques have identified candidate pathogenic genes in PSIS patients. Notably, some gene mutations are closely associated with pituitary development, including NR0B1, BMP2, BMP4, FGF8, GATA2, GLI1, GLI2, RBPJ, PCSK1, POU1F1, PROP1, NKX2-1, WNT5A, HESX1, WNT4, and TBX19 (**Supplementary Table 1**). The detection of genetic alterations in these genes provides insights into the molecular mechanisms underlying pituitary gland development and suggests their potential role in the pathogenesis of PSIS. Fourthly, approximately 20-50% of PSIS patients exhibit concurrent developmental abnormalities in midline structures of the central nervous system (9, 16, 20). These abnormalities can include conditions such as cleft lip, optic nerve hypoplasia, partial agenesis of the corpus callosum, spina bifida, and encephalocele. The coexistence of these midline structural malformations further supports the involvement of genetic defects affecting the development of the central nervous system. Lastly, PSIS patients often present with extra-pituitary malformations, such as micropenis, cryptorchidism, and Fanconi anemia. Of note, limb malformations have been reported in approximately 9%-20% of PSIS patients (9, 16, 20). The presence of these associated malformations provides additional evidence for the involvement of genetic abnormalities that affect multiple organ systems.

These conflicting pieces of evidence highlight the complex nature of PSIS etiology, suggesting that the pathogenesis is likely influenced by a combination of perinatal factors and congenital genetic defects, rather than being solely attributable to perinatal injuries or congenital genetic defects. Further investigations are warranted to unravel the intricate mechanisms underlying PSIS and to elucidate the interplay between genetic predisposition and environmental factors in PSIS pathogenesis.

### 4.2 Current status of genetic research on PSIS patients

The investigation of pathogenic genes in PSIS patients has been a hot topic in recent years, leading to numerous related literature reports on this topic. In our comprehensive literature search, we identified a total of 602 articles related to PSIS, of which 37 studies specifically focused on gene sequencing in PSIS patients. Additionally, we found six reports that explored chromosomal abnormalities in PSIS patients. Among 6 familial cases, mutations in the ROBO1 gene were found in 3 families. However, it is important to note that these studies did not undertake subsequent *in vitro* or *in vivo* functional validation of the identified ROBO1 mutations. In another familial case, both sisters diagnosed with PSIS were found to carry the PROKR2 A51T mutation. Nevertheless, *in vitro* functional validation experiments revealed that the PROKR2 A51T mutation did not significantly impact receptor activity (42). Similarly, in a different familial case, both siblings diagnosed with PSIS were found to harbor the LHX4: c.607-1G>C mutation. Subsequent *in vitro* investigations demonstrated abnormal splicing of LHX4 PCR products resulting from this mutation (46). Lastly, in another familial case, both sisters diagnosed with PSIS were found to carry the GPR161: c.56T>A, p.L19G mutation (38). However, the study did not conduct subsequent functional validation experiments to assess the functional implications of this mutation. It is crucial to recognize that these findings alone do not establish a direct causal relationship between the mutation and the occurrence of PSIS in the affected patients.

In the 37 articles included in our analysis, the majority of studies primarily relied on bioinformatics approaches to evaluate the pathogenicity of gene mutations and assess their impact on protein structure and function. Only 10 articles conducted subsequent functional validation of the identified gene mutations using cellular or animal models, two of which have been already discussed in the preceding text. Emily et al. conducted exon sequencing in six PSIS patients and identified seven variations in FAT2 and DCHS2 (22). All patients exhibited growth hormone deficiency, with two cases displaying multiple hormone deficiencies and a smaller pituitary gland. In *Dchs2* ^-/-^ mutant mice, the researchers observed anterior pituitary dysplasia and partial penetrance of the sellar floor defect. Similarly, *Fat4* ^-/-^ and *Dchs1* ^-/-^ mutant mice exhibited sellar floor abnormalities and significant defects in anterior pituitary morphology. However, it is worth noting that the cell types of the anterior pituitary in these three gene knockout mouse models appeared normal, which does not fully align with the clinical manifestations observed in the six PSIS cases. In another study conducted by Shana et al., whole-exome sequencing identified heterozygous missense mutations in PROKR2 and WDR11 in a single PSIS patient (35). The PROKR2 mutation (c.253C>T, p.R85C) was inherited from the unaffected mother, while the WDR11 mutation (c.1306A>G, p.I436V) was inherited from the unaffected father. Through various experimental techniques, such as mutant plasmid construction, cell transfection, co-immunoprecipitation, and immunofluorescence, Shana et al. validated that the mutant WDR11 protein lost its ability to bind with EMX1, resulting in its inability to pass through the nuclear membrane. Bando et al. uncovered SIX3 and POU1F1 double heterozygous mutations in two PSIS patients (15). They validated that disrupting *Six3* expression in the oral ectoderm led to complete ablation of anterior pituitary development, while deleting *Six3* in the neural ectoderm hindered the development of the pituitary stalk, along with both anterior and posterior pituitary lobes, using conditional gene knockout mouse models. Gregory et al. reported a PSIS patient with the heterozygous variant OTX2: c.689A>T, p.H230L. Nonetheless, findings from dual-luciferase reporter assays and *Otx2* ^H230L/+^ mice suggest that this variant retains transactivation properties and does not significantly impact pituitary or eye development of mice (17). Kaygusuz et al. described a novel FOXA2: c.616C >T, p.Q206X variant in one PSIS patient, causing impaired GLUT2-luciferase activation due to a truncated protein (19). Wang et al. observed a significant reduction in LHX3 expression when NBPF9 was knocked down in human embryonic stem cells (25). Su et al. found that silencing the CDC27 gene inhibits both migration and secretion functions in GH3 cells. While these findings offer valuable insights into potential genetic factors associated with PSIS, the lack of comprehensive functional validation in some cases underscores the need for further investigations to ascertain the functional significance and causal relationship between these mutations and PSIS. Robust experimental studies, including *in vitro* and *in vivo* assays, are necessary to unravel the precise molecular mechanisms underlying the pathogenesis of PSIS and to establish a definitive understanding of the contribution of these identified mutations to PSIS.

It is important to highlight that the majority of research focused on gene sequencing in PSIS patients has primarily been confined to the identification of potential pathogenic mutations using high-throughput sequencing technology and subsequent bioinformatics analyses. While these approaches have helped identify candidate genes enriched in signaling pathways associated with pituitary and neural development, thorough functional studies and animal models are still lacking to validate these new candidate genes and explore their potential interactions. Existing evidence is insufficient to fully elucidate the complex pathogenesis of PSIS. Therefore, continuous efforts are needed to develop novel methods and tools that can aid in the interpretation and validation of the vast amount of data generated by gene sequencing, ultimately advancing our understanding of PSIS.

### 4.3 Our gene analysis based on literature

The utilization of high-throughput technologies, such as whole-exome sequencing and whole-genome sequencing, has significantly facilitated the identification of a vast number of potential candidate gene mutations associated with PSIS. Nonetheless, a common challenge encountered in gene studies utilizing these high-throughput technologies is the limited identification of single or relatively concentrated mutations. The presence of multiple mutated genes suggests that PSIS is a genetically heterogeneous disorder rather than a monogenic disease. Thus, at present, there is no clear pattern linking the identified genes to the pathogenesis of PSIS. In our previous sequencing studies, we observed that candidate pathogenic mutations were predominantly enriched in three signaling pathways: the Notch signaling pathway, Wnt signaling pathway, and Hedgehog signaling pathway (10). To further explore the landscape of genes associated with PSIS, we conducted a comprehensive review of all reported genes related to PSIS from both domestic and international sources. After eliminating duplicate records, we obtained a total of 224 mutated genes (**Supplementary Table 2**). The KEGG enrichment analysis performed on these 224 mutated genes revealed their significant enrichment in several signaling pathways, including the Notch signaling pathway, Wnt signaling pathway, Hedgehog signaling pathway, Signaling pathways regulating pluripotency of stem cells, and Axon guidance. These signaling pathways play pivotal roles in regulating the development of the hypothalamus-pituitary axis, which is integral to the pathogenesis of PSIS (56–59). Furthermore, a subsequent GO Biological Processes analysis demonstrated that these genes were primarily enriched in fundamental biological processes, such as embryonic development, organogenesis (including the development of key organs like the brain, pituitary gland, hypothalamus, heart, and kidneys), as well as axon generation, development, guidance, and function. Although the highly pathogenic candidate genes identified in the aforementioned literature reports may not possess direct relevance to PSIS, the results obtained from the enrichment analysis indicated a certain degree of correlation between these genes and the development of critical structures like the pituitary gland, hypothalamus, and the anterior or intermediate regions of the forebrain.

In individuals diagnosed with PSIS, there is a higher prevalence of breech presentation during delivery, however, the underlying relationship between the increased rate of breech presentation and gene mutations remains elusive. Some investigations propose that gene mutations and congenital abnormalities in the development of the forebrain or pituitary gland could potentially contribute to breech presentation (60–62). A study utilizing whole-exome sequencing in patients with isolated ectopic posterior pituitary (EPP) revealed a significantly elevated frequency of breech presentation in the subgroup with identified gene mutations compared to EPP patients without detected gene mutations (5/15 vs. 1/63; Z-test, P = 0.003) (16). Although these findings are based on limited data, they suggest a potential correlation between gene mutations and adverse perinatal events such as breech presentation. Moreover, it has been observed that children with isolated growth hormone deficiency, a condition associated with congenital pituitary dysfunction, exhibit a higher incidence of perinatal injuries (40). Large-scale data analyses have also demonstrated an increased occurrence of breech presentation in cases of congenital neurodevelopmental abnormalities (63). Conditions such as congenital double pituitary, septo-optic dysplasia (SOD), and idiopathic growth hormone deficiency have been linked to a higher prevalence of breech presentation and challenging labor (64, 65). These pieces of evidence provide support for the notion that gene mutations leading to congenital abnormalities in the development of the forebrain or pituitary gland may contribute to abnormal breech presentation during delivery. Therefore, it can be inferred that gene mutations associated with the development of the forebrain or pituitary gland may play a role in the occurrence of breech presentation in individuals with PSIS. However, further research is warranted to elucidate the specific genetic mechanisms underlying this relationship and to provide a more comprehensive understanding of the complex interplay between gene mutations and breech presentation in PSIS patients.

From an embryonic development perspective, the pituitary gland undergoes intricate processes to form its mature structure. It arises from the upward extension of the oral ectoderm and the downward extension of the hypothalamic neuroectoderm, eventually culminating in the formation of the anterior and posterior lobes within the sella turcica (66–70). However, in the context of PSIS, the posterior lobe exhibits ectopic locations that vary among affected individuals, ranging from a high position near the infundibular recess to a low position closer to the pituitary gland (71, 72). Notably, the ectopic positioning of the posterior lobe follows the path of the pituitary stalk. This phenomenon could be attributed to varying degrees of traction and damage exerted on the hypothalamic-pituitary region during delivery or different genetic mutations affecting the development and neural migration of the hypothalamic-pituitary region (73, 74). In a reported case of a pair of twins, the firstborn twin diagnosed with PSIS and posterior lobe ectopia experienced breech presentation and difficult labor, while the second twin, without any perinatal events, did not exhibit posterior lobe ectopia or PSIS (29). These findings strongly suggest that the potential injury secondary to traction during delivery may be a direct cause of PSIS (75). Regardless of fetal position, if there is sufficient traction during delivery, it can lead to upward retraction of the extending posterior lobe. Additionally, patients born in the breech position are more prone to perinatal events, which increase the likelihood of traction during delivery and the occurrence of PSIS. This elucidates why both breech and normally positioned individuals may experience posterior lobe ectopia. Indeed, individuals born in the breech position have a higher proportion of PSIS compared to those born in the normal position due to a higher likelihood of difficult labor and traction. Of note, even with normal fetal position, if there is sufficient traction during delivery, it can also lead to upward retraction of the extending posterior lobe. Therefore, both breech and normally positioned individuals may experience posterior lobe ectopia. Various gene defects may affect fetal position, increasing the risk of difficult labor and potentially resulting in increased traction, which further increases the risk and severity of posterior lobe ectopia. Traction, which can occur in both breech (higher risk of genetic defects) and normal (lower risk of genetic defects) fetal positions, adds another layer of complexity to the risk of PSIS. This complexity may contribute to the uncertainty and irregularity in the relationship between detected candidate gene defects and PSIS.

### 4.4 Our new hypothesis

**Figure 4:**
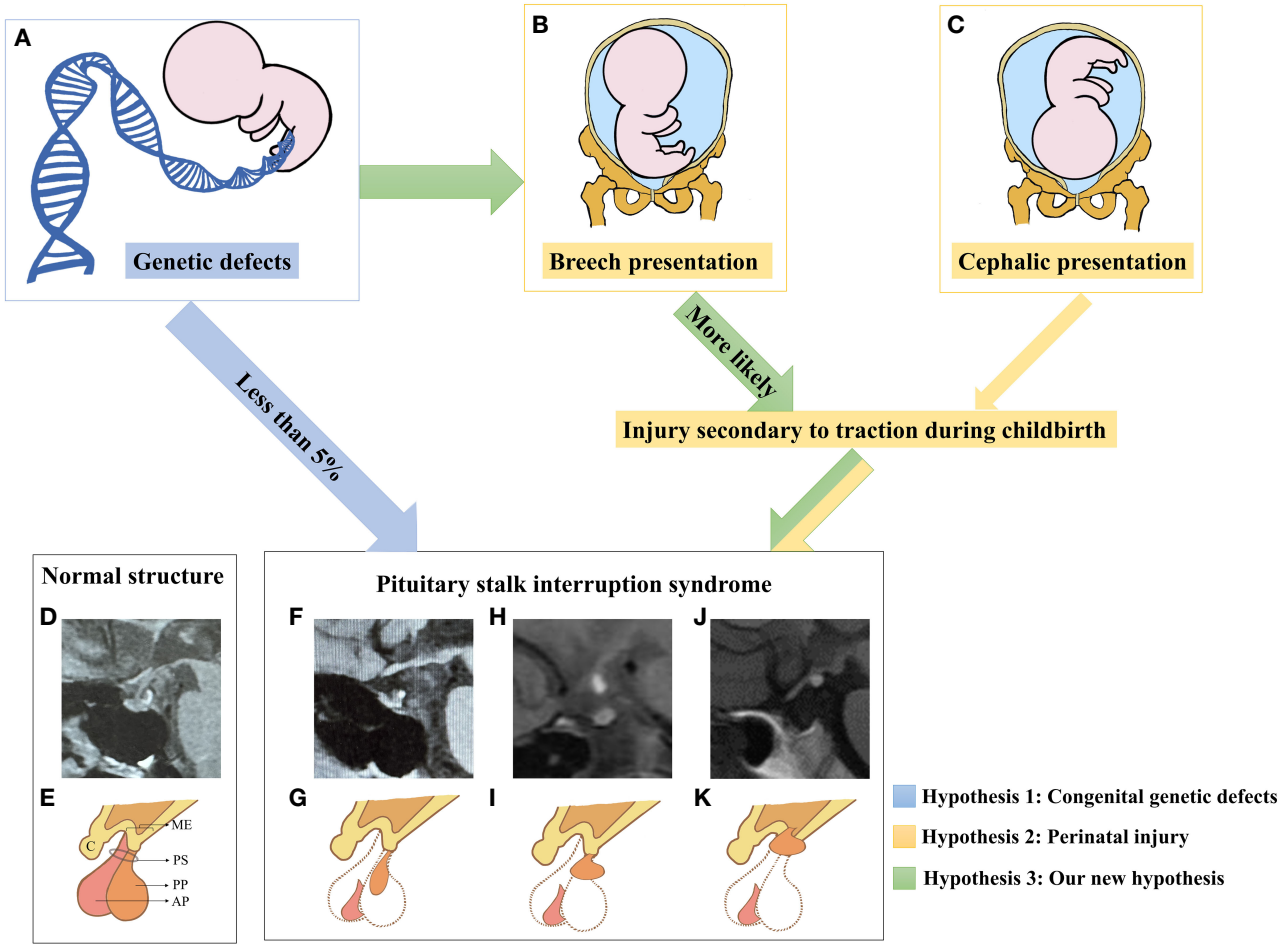
A new etiological hypothesis of PSIS. **(A–C)** Pathogenic genetic defects were identified in only less than 5% of PSIS patients. In the existing reports, mutated genes in PSIS patients were associated with the development of the pituitary gland, hypothalamus, and the anterior or intermediate regions of the forebrain, which may increase the rate of perinatal adverse events such as breech presentation and neonatal distress. Compared with newborns who underwent normal delivery, newborns who experienced perinatal adverse events were more likely to suffer more injuries secondary to traction during childbirth. Different degrees of damage to the hypothalamic-pituitary region caused by traction may be an important factor for different locations of ectopia posterior lobe in PSIS patients. **(D)** MRI image of the normal hypothalamic-pituitary region. **(E)** Illustrative diagram corresponding to D. ME, median eminence; C, chiasm; PS, pituitary stalk; AP, anterior pituitary; PP, posterior pituitary. **(F)** The MRI image shows anterior pituitary hypoplasia, an invisible pituitary stalk, and ectopic location of the posterior pituitary gland near the pituitary fossa along the PS. **(G)** Illustrative diagram corresponding to **(F)**. **(H)** The MRI image shows ectopic location of the posterior pituitary gland in the middle of PS. **(I)** Illustrative diagram corresponding to **(H)**. **(J)** The MRI image shows anterior pituitary hypoplasia, an invisible pituitary stalk, and ectopic location of the posterior pituitary gland in the ME. **(K)** Illustrative diagram corresponding to **(H)**. To obtain additional magnetic resonance images of pituitary posterior lobe ectopia in different locations, please refer to this review article (71).

Based on the analysis above, our hypothesis (**Figure 4**) regarding the pathogenesis of PSIS posits that the genetic background of individuals and/or the presence of specific gene mutations can disrupt normal embryonic development, resulting in forebrain abnormalities and/or abnormalities in the development of the hypothalamic-pituitary region. As a consequence, this can lead to an increased proportion of abnormal fetal deliveries, and the varying degrees of traction experienced during these abnormal deliveries may contribute to different degrees of pituitary stalk interruption and ectopia posterior lobe. In cases where pituitary stalk development is imperfect, hypothalamic-releasing hormones are unable to properly reach the pituitary gland, causing progressive atrophy of pituitary cells that lack adequate hypothalamic stimulation (76). This presents as the characteristic triad of posterior lobe ectopia, pituitary stalk absence or interruption, and anterior lobe hypoplasia. In other words, our hypothesis posits that the pathogenesis of PSIS arises from the combined effect of congenital gene defects and mechanical forces exerted during delivery at least in some PSIS patients (77). The genetic background of individuals with PSIS, along with the predisposed specific gene mutations and the varying degrees of mechanical forces during delivery, are important factors contributing to the clinical heterogeneity observed in PSIS cases.

## Data availability statement

The datasets presented in this study can be found in online repositories. The names of the repository/repositories and accession number(s) can be found in the article/**Supplementary Material**.

## Ethics statement

Ethical approval was not required for this study, since this article was further in-depth analysis based on already published data.

## Author contributions

SW: Conceptualization, Data curation, Formal analysis, Visualization, Writing – original draft. QQ: Data curation, Investigation, Writing – review & editing. DJ: Investigation, Validation, Writing – review & editing. YX: Investigation, Methodology, Writing – review & editing. LY: Investigation, Writing – review & editing. XJ: Project administration, Writing – review & editing. QG: Funding acquisition, Supervision, Writing – review & editing.
