# Supplementary material for: Re-analysis of gene mutations found in pituitary stalk interruption syndrome and a new hypothesis on the etiology
Source: Front Endocrinol (Lausanne). 2024 Feb 23;15:1338781. doi: 10.3389/fendo.2024.1338781 (PMC10920343; doi:10.3389/fendo.2024.1338781)
Supplement: Supplementary file 1 [file Table_1.docx]

Supplemental Table 1: The detailed genes enriched in various pathways and processes that match Figure 2D-G

| Description | enrichment | p-value | count | genes |
| --- | --- | --- | --- | --- |
| Notch signaling pathway | 0.288136 | 1E-22 | 17 | JAG1, CREBBP, CTBP2, DVL1, RBPJ, LFNG, NOTCH2, NOTCH3, PSEN1, PSEN2, ATXN1, ADAM17, NUMBL, NCOR2, DLL1, DLL4, MAML3 |
| Wnt signaling pathway | 0.129412 | 1E-20 | 22 | CREBBP, CTBP2, DVL1, LRP6, MMP7, MYC, NFATC1, NFATC2, PLCB2, PSEN1, WNT5A, AXIN1, FZD1, FZD8, CER1, ROCK2, FRAT1, DAAM1, WNT4, NKD2, PRICKLE2, CCDC88C |
| Hedgehog signaling pathway | 0.232143 | 1E-16 | 13 | GLI1, GLI2, GLI3, LRP2, PTCH1, SHH, SMO, PTCH2, GPR161, CDON, HHAT, HHIP, KIF7 |
| Signaling pathways regulating pluripotency of stem cells | 0.111888 | 1E-14 | 16 | BMP4, DVL1, FGFR1, FGFR3, FGFR2, MYC, OTX1, MAPK3, RAF1, WNT5A, KAT6A, AXIN1, FZD1, FZD8, HESX1, WNT4 |
| Axon guidance | 0.082418 | 1E-11 | 15 | CDK5, NFATC2, MAPK3, PTCH1, RAF1, ROBO1, ROBO2, SHH, SMO, WNT5A, SLIT2, ROCK2, SEMA3E, SEMA3A, WNT4 |
| Hippo signaling pathway | 0.082803 | 2E-10 | 13 | BMP2, BMP4, BMP6, BMP8B, DVL1, GLI2, MYC, WNT5A, AXIN1, FZD1, FZD8, WNT4, NKD2 |
| cAMP signaling pathway | 0.062222 | 1.58E-09 | 14 | CFTR, CREBBP, FSHR, GLI1, GLI3, GNAS, NFATC1, POMC, MAPK3, PTCH1, RAF1, VIPR2, ROCK2, HHIP |
| TGF-beta signaling pathway | 0.094737 | 3.98E-08 | 9 | BMP2, BMP4, BMP6, BMP8B, CREBBP, IGSF1, MYC, MAPK3, TGIF1 |
| PI3K-Akt signaling pathway | 0.039548 | 5.01E-07 | 14 | COL1A1, COL1A2, FGF8, FGFR1, FGFR3, FGFR2, GH1, MYC, PDGFRB, PPP2R5B, PPP2R5D, MAPK3, RELN, RAF1 |
| MAPK signaling pathway | 0.037415 | 1.26E-05 | 11 | FGF8, FGFR1, FGFR3, FGFR2, MYC, NF1, NFATC1, NFKB2, PDGFRB, MAPK3, RAF1 |
| mTOR signaling pathway | 0.051282 | 2.51E-05 | 8 | DVL1, LRP6, MAPK3, RAF1, WNT5A, FZD1, FZD8, WNT4 |
| cGMP-PKG signaling pathway | 0.041916 | 0.000251 | 7 | NFATC1, NFATC2, PLCB2, MAPK3, RAF1, ROCK2, KCNMB2 |
|  |  |  |  |  |
| tissue morphogenesis | 0.091873 | 1E-41 | 52 | JAG1, AR, BMP2, BMP4, DVL1, FGF8, FGFR2, GLI2, GLI3, RBPJ, IL10, LHX1, LRP2, MSX1, MTHFR, MYC, NOTCH2, NPHP1, PKD1, PKD2, PSEN1, PTCH1, ROBO1, ROBO2, SHH, STIL, SIX3, SMO, SOX11, ADAM17, TBX2, NKX2-1, WNT5A, WT1, AXIN1, EOMES, FZD1, DCHS1, HESX1, TBX19, SLIT2, SEMA3E, DLL1, WNT4, DLL4, AHI1, CHD7, CC2D2A, HHIP, TCTN1, CEP290, GATA5 |
| embryonic morphogenesis | 0.089501 | 1E-40 | 52 | NR0B1, BMP4, FOXL2, CREBBP, DVL1, FGF8, FGFR2, GATA2, GLI1, GLI2, GLI3, FOXA2, IL10, LHX1, LRP2, MSX1, MTHFR, NOTCH2, OTX1, OTX2, PKD2, MAPK3, PSEN1, PTCH1, SHH, STIL, SIX3, SMO, SOX11, TBX2, WNT5A, AXIN1, EOMES, FZD1, HESX1, TBX19, CER1, ROCK2, IFT140, B9D1, DLL1, CDON, SIX4, WNT4, DLL4, AHI1, CHD7, CC2D2A, CDH23, TCTN1, CEP290, TBC1D32 |
| embryonic organ development | 0.084444 | 1E-28 | 38 | BMP4, FOXL2, DVL1, FGF8, FGFR2, GATA2, GLI1, GLI2, GLI3, RBPJ, IL10, LHX1, KMT2A, MSX1, NOTCH2, OTX1, PKD1, PKD2, MAPK3, PSEN1, PTCH1, SHH, STIL, SIX3, SMO, SOX11, TBX2, WNT5A, EOMES, HESX1, IFT140, DLL1, SIX4, AHI1, CHD7, CC2D2A, CDH23, CEP290 |
| dorsal/ventral pattern formation | 0.223529 | 1E-22 | 19 | BMP4, FGF8, GLI1, GLI2, GLI3, LHX1, PROP1, PSEN1, PTCH1, SHH, SIX3, SMO, NKX2-1, AXIN1, CER1, DLL4, HHIP, TCTN1, TBC1D32 |
| forebrain development | 0.126263 | 1E-46 | 50 | NR0B1, BMP2, BMP4, CDK5, FGF8, FGFR2, GATA2, GLI1, GLI2, GLI3, RBPJ, LHX1, LRP2, MSX1, NF1, NOTCH3, OTX1, OTX2, PCSK1, POU1F1, PROP1, RELN, PSEN1, ROBO1, ROBO2, SHH, STIL, SIX3, SMO, NKX2-1, WNT5A, EOMES, HESX1, TBX19, NUMBL, SLIT2, SEMA3E, KIF14, SEMA3A, DISC1, CDON, NIN, WNT4, NDE1, CHD7, ZSWIM6, TCTN1, CEP120, ASPM, CCDC141 |
| telencephalon development | 0.114815 | 1E-27 | 31 | BMP2, BMP4, CDK5, FGF8, GLI3, LHX1, NF1, RELN, PSEN1, ROBO1, ROBO2, SHH, SIX3, SMO, NKX2-1, WNT5A, EOMES, NUMBL, SLIT2, KIF14, SEMA3A, DISC1, CDON, NIN, NDE1, CHD7, ZSWIM6, TCTN1, CEP120, ASPM, CCDC141 |
| diencephalon development | 0.272727 | 1E-27 | 21 | NR0B1, BMP2, BMP4, FGF8, GATA2, GLI1, GLI2, RBPJ, OTX1, PCSK1, POU1F1, PROP1, SHH, SIX3, SMO, NKX2-1, WNT5A, HESX1, TBX19, SEMA3E, WNT4 |
| pituitary gland development | 0.404762 | 1E-25 | 17 | NR0B1, BMP2, BMP4, FGF8, GATA2, GLI1, GLI2, RBPJ, PCSK1, POU1F1, PROP1, SIX3, NKX2-1, WNT5A, HESX1, TBX19, WNT4 |
| hypothalamus development | 0.153846 | 3.98E-05 | 4 | NR0B1, PROP1, NKX2-1, SEMA3E |
| axonogenesis | 0.083573 | 1E-21 | 29 | CDK5, DVL1, FGFR2, GLI2, GLI3, LHX1, NOTCH2, NOTCH3, OTX2, RELN, PTCH1, ROBO1, ROBO2, SHH, SMO, NKX2-1, WNT5A, USP9X, NUMBL, SLIT2, SEMA3E, SEMA3A, TUBB3, NIN, LHX9, TCTN1, SPG11, LHX4, CRPPA |
| axon development | 0.074742 | 1E-20 | 29 | CDK5, DVL1, FGFR2, GLI2, GLI3, LHX1, NOTCH2, NOTCH3, OTX2, RELN, PTCH1, ROBO1, ROBO2, SHH, SMO, NKX2-1, WNT5A, USP9X, NUMBL, SLIT2, SEMA3E, SEMA3A, TUBB3, NIN, LHX9, TCTN1, SPG11, LHX4, CRPPA |
| neuron projection guidance | 0.106481 | 1E-19 | 23 | CDK5, DVL1, GLI2, GLI3, LHX1, NOTCH2, NOTCH3, OTX2, RELN, PTCH1, ROBO1, ROBO2, SHH, SMO, NKX2-1, WNT5A, SLIT2, SEMA3E, SEMA3A, TUBB3, LHX9, LHX4, CRPPA |
| axon guidance | 0.106481 | 1E-19 | 23 | CDK5, DVL1, GLI2, GLI3, LHX1, NOTCH2, NOTCH3, OTX2, RELN, PTCH1, ROBO1, ROBO2, SHH, SMO, NKX2-1, WNT5A, SLIT2, SEMA3E, SEMA3A, TUBB3, LHX9, LHX4, CRPPA |
| heart development | 0.080071 | 1E-32 | 45 | JAG1, BMP2, BMP4, DVL1, FGF8, FGFR2, GLI2, GLI3, RBPJ, LRP2, MSX1, NF1, NFATC1, NOTCH2, PDGFRB, PKD1, PKD2, MAPK3, PSEN1, PTCH1, ROBO1, ROBO2, SHH, STIL, SMO, SOX11, TBX2, WNT5A, WT1, EOMES, FZD1, DCHS1, TBX19, CER1, SLIT2, ROCK2, IFT140, DLL1, DLL4, AHI1, CHD7, WDR11, CC2D2A, GATA5, TBC1D32 |
| kidney development | 0.118644 | 1E-31 | 35 | JAG1, BMP2, BMP4, BMP6, FGF8, FGFR2, GLI2, GLI3, LHX1, LRP2, MYC, NF1, NOTCH2, NOTCH3, PDGFRB, PKD1, PKD2, PTCH1, ROBO2, SHH, SMO, SOX11, WNT5A, WT1, DCHS1, CER1, SLIT2, DLL1, SIX4, WNT4, DCHS2, AHI1, CC2D2A, CEP290, TBC1D32 |
|  |  |  |  |  |
| ciliary base | 0.148936 | 5.01E-08 | 7 | GLI1, GLI2, GLI3, PRKAR2A, PRKAR2B, DISC1, TTC26 |
| ciliary tip | 0.145833 | 6.31E-08 | 7 | GLI1, GLI2, GLI3, SMO, IFT140, TTC26, KIF7 |
| ciliary transition zone | 0.098592 | 0.000001 | 7 | NPHP1, IFT140, B9D1, AHI1, CC2D2A, TCTN1, CEP290 |
| ciliary basal body | 0.071856 | 5.01E-09 | 12 | PKD2, IFT140, DAAM1, B9D1, DISC1, AHI1, WDR11, CENPJ, TTC26, CEP290, CEP41, KIF7 |
| cilium | 0.047684 | 1E-18 | 35 | GLI1, GLI2, GLI3, NOTCH2, NPHP1, PKD1, PKD2, PRKAR2A, PRKAR2B, PSEN1, PSEN2, PTCH1, SMO, TACR3, USP9X, IFT140, DAAM1, GPR161, B9D1, DISC1, NIN, AHI1, WDR11, CENPJ, INPP5E, CC2D2A, CDH23, HHIP, TCTN1, TTC26, CEP290, KISS1R, CEP41, TBC1D32, KIF7 |
| distal axon | 0.043321 | 1.26E-06 | 12 | CAD, CDK5, DVL1, OTX2, PCSK1, PSEN1, PSEN2, PTCH1, USP9X, TUBB3, DISC1, NIN |
| axon | 0.030016 | 3.16E-07 | 19 | CAD, CDK5, DVL1, LRP2, NF1, OTX2, PCSK1, PSEN1, PSEN2, PTCH1, ROBO1, ROBO2, USP9X, SLC12A6, SEMA3A, TUBB3, DISC1, NIN, SPG11 |
| dendrite | 0.029752 | 7.94E-07 | 18 | AVP, CDK5, DVL1, LRP2, NF1, PCSK1, PRKAR2B, RELN, PSEN1, PSEN2, PTCH1, SMO, TACR3, SEMA3A, TUBB3, NSMF, NIN, SPG11 |
| neuronal cell body | 0.024 | 0.000398 | 12 | CAD, CDK5, DVL1, LRP6, PCSK1, PMM2, PRKAR2B, PSEN1, PSEN2, TACR3, TUBB3, NSMF |
| transcription regulator complex | 0.040777 | 3.16E-10 | 21 | CHD4, CREBBP, CTBP2, GATA2, GLI3, RBPJ, LHX1, MSX1, MYC, NFATC1, NFATC2, SIX6, PROP1, ATXN7, SIX3, TBX2, NKX2-1, NCOR2, ARNT2, SIX4, SIX5 |
| transcription repressor complex | 0.064103 | 0.000316 | 5 | CTBP2, GLI3, RBPJ, MYC, NCOR2 |
| receptor complex | 0.022472 | 0.000631 | 12 | BMP2, FGFR1, FGFR3, FGFR2, FSHR, GH1, LRP2, NOTCH2, NOTCH3, PDGFRB, ARNT2, GPR101 |
| extracellular matrix | 0.021622 | 0.001 | 12 | COL1A1, COL1A2, FGFR2, GH1, MMP7, MUC4, RELN, SHH, WNT5A, CDON, WNT4, FREM1 |
|  |  |  |  |  |
| hedgehog receptor activity | 1 | 3.98E-07 | 3 | PTCH1, PTCH2, B9D1 |
| fibroblast growth factor receptor activity | 0.6 | 3.98E-06 | 3 | FGFR1, FGFR3, FGFR2 |
| BMP receptor binding | 0.3 | 5.01E-05 | 3 | BMP2, BMP4, BMP6 |
| Wnt receptor activity | 0.25 | 5.01E-06 | 4 | LRP6, PKD1, FZD1, FZD8 |
| Notch binding | 0.192308 | 1.26E-06 | 5 | JAG1, ADAM17, NCOR2, DLL1, DLL4 |
| DNA-binding transcription activator activity | 0.050847 | 1E-13 | 24 | AR, FOXL2, EGR4, ELF4, GATA2, GLI1, RBPJ, MSX1, MYC, NFATC1, NFATC2, NFKB2, OTX1, OTX2, POU1F1, PROP1, SIX3, SOX11, WT1, TBX19, ARNT2, SIX4, LHX4, SIX5 |
| transcription factor binding | 0.040067 | 1E-11 | 24 | NR0B1, AR, FOXL2, CHD4, CREBBP, CTBP2, GATA2, RBPJ, MYC, NFATC1, NFATC2, NOTCH2, POU1F1, MAPK3, SIX3, TBX2, NKX2-1, KAT6A, EOMES, NCOR2, ARNT2, STK36, DLL1, NSD1 |
| DNA-binding transcription factor binding | 0.043933 | 1E-10 | 21 | NR0B1, AR, FOXL2, CHD4, CREBBP, CTBP2, GATA2, RBPJ, MYC, NFATC1, NOTCH2, POU1F1, MAPK3, TBX2, NKX2-1, KAT6A, EOMES, NCOR2, ARNT2, DLL1, NSD1 |
| signaling receptor regulator activity | 0.036101 | 7.94E-09 | 20 | JAG1, AVP, BMP2, BMP4, BMP6, BMP8B, CDK5, FGF8, GH1, IGSF1, IL10, POMC, SHH, WNT5A, CER1, SEMA3E, SEMA3A, DLL1, WNT4, DLL4 |
| G protein-coupled receptor binding | 0.041522 | 2E-06 | 12 | AVP, DVL1, GNAS, LRP6, POMC, PTCH1, WNT5A, FZD1, PTCH2, WNT4, PROK2, CCDC88C |
